# Expanding access to HIV prevention

**DOI:** 10.1186/1742-6405-3-2

**Published:** 2006-01-17

**Authors:** Helene D Gayle

**Affiliations:** 1 Director, HIV, TB, and Reproductive Health, Bill & Melinda Gates Foundation

## Background

The expanding HIV/AIDS epidemic represents one of the greatest threats to human health and international development today, and strengthening the global response is imperative. Despite recent progress in expanding access to HIV/AIDS treatment [1], the world continues to severely under-invest in efforts to fight HIV/AIDS, missing a tremendous opportunity to change the course of the epidemic by bringing proven treatment and prevention interventions to scale.

Nowhere is the need for an expanded response more apparent – and the potential impact greater – than in efforts to prevent the spread of new infections. In 2005 alone, nearly 5 million people worldwide became infected with HIV, and today more than 40 million people are living with HIV, more than in any previous year [2]. Although extensive studies have shown the effectiveness of numerous strategies for preventing the transmission of HIV [3], new infections continue to occur at alarming rates because proven tools are not being used. Fewer than one in five people at high risk for HIV have access to effective prevention [4], and funding for prevention programs is woefully inadequate [5]. There is also an urgent need to increase research funding and accelerate the development of new technologies for HIV prevention [6].

By bringing new and more focused attention to prevention, the world can slow, and ultimately reverse, the spread of HIV. An analysis in 2002 by the World Health Organization and UNAIDS found that expanded access to existing prevention strategies could avert up to two-thirds of the 45 million HIV infections projected to occur between 2002 and 2010 [7], and a study in early 2005 affirmed that expanded access to these tools could stop roughly half of infections by 2020 [8]. Still more infections could be prevented by the development and introduction of new tools currently being evaluated, such as topical microbicides, new treatments for other sexually transmitted diseases, male circumcision, female diaphragms, and the best long-term hope, a preventive vaccine.

Expanded access to prevention is also critical for sustaining the important progress being made in providing antiretroviral therapy to people living with HIV. Unless the rate of new infections is sharply curtailed, treatment will never be able to keep pace with the growing need. For example, while the World Health Organization's recent "3 by 5" campaign sought to provide antiretroviral treatment to 3 million people by 2005, 5 million people became newly infected this year alone.

To help accelerate HIV prevention access and research, the Bill & Melinda Gates Foundation's Global Health program has committed approximately $1.1 billion in grants for HIV/AIDS programs since 1998 [9]. HIV/AIDS is among the priority diseases and health conditions addressed by the foundation, which provides support to organizations worldwide to address health problems that cause the greatest illness and death in developing countries, yet receive far too little attention and resources. The remainder of this article describes the foundation's HIV/AIDS grantmaking priorities, which focus on two critical areas: first, maximizing use of currently available prevention tools and integrating them with treatment and care; and second, accelerating research on new prevention tools.

## Maximizing available tools

A top priority for the foundation is expanding access to proven HIV prevention strategies for those who need them most. In Botswana, where an estimated 35% of the adult population is living with HIV infection [10], the foundation is partnering with the national government and Merck & Co., Inc., to bring a comprehensive prevention and treatment response to national scale. In India, the foundation's $200 million Avahan initiative aims to expand access to HIV prevention programs for the most vulnerable populations, including sex workers, their clients, injection drug users, and migrant workers. Avahan works in partnership with national and local governments and NGOs, and every month reaches tens of thousands of people in six high-prevalence states and along the most-traveled highways.

The foundation's programs to expand access to HIV prevention are large-scale demonstration projects designed to show what is possible and catalyze others to provide support. Our resources, though significant, are not great enough to solve countries' HIV/AIDS challenges, and sustaining access to health services must ultimately be the primary responsibility of the public sector. For example, the foundation's contribution of $150 million to the Global Fund to Fight AIDS, Tuberculosis, and Malaria – which is assisting 128 countries in scaling up national responses to HIV/AIDS and other leading infectious diseases – was intended to help the fund get started and attract other donors.

As HIV prevention programs are scaled up, it is essential that they make use of the full range of available prevention options, and that prevention programs are closely integrated with treatment services. The foundation is a co-convener of the Global HIV Prevention Working Group, an international body of HIV/AIDS experts focused on HIV prevention analysis and advocacy. In 2004, the Working Group released *HIV Prevention in the Era of Expanded Treatment Access*, the first report to identify the benefits and challenges to effective prevention posed by expanding treatment access. (See .)

## Accelerating research on new tools

While today's tools have been proven effective, there is no "magic bullet" for HIV prevention. Women, in particular, need far greater options to protect themselves from infection. The foundation has committed substantial funds to research new prevention technologies that could have a substantial impact on reducing new HIV infections, including many that could be initiated by women. The foundation provides grants to support basic research and clinical trials of investigational HIV prevention technologies, some of which could be available within the next few years:

### Microbicides

Microbicides are topical gels or creams designed to be applied to the vagina or rectum to prevent HIV infection. The foundation has made grants to support a variety of research and development efforts for microbicides, including support for a large-scale efficacy trial of a leading microbicide candidate.

### Herpes treatment for HIV prevention

Like many other sexually transmitted diseases, herpes (HSV-2) infection significantly increases the risk of HIV transmission. The foundation is supporting clinical research to assess whether using the inexpensive drug acyclovir to treat HSV-2 reduces the risk of HIV acquisition and transmission.

### Diaphragms

Most HIV infections in women are believed to occur in the cervix and endocervix. By covering the cervix, the female diaphragm may provide protection against HIV infection. The foundation has provided grants to support studies to help assess diaphragms for HIV prevention in developing countries.

### Male circumcision

Results from an initial clinical trial in South Africa suggest that male circumcision reduces the likelihood of female-to-male sexual HIV transmission by 60% [11]. In order to confirm these results, the foundation is supporting a trial in Uganda, which is designed to determine if male circumcision also reduces the risk of male-to-female HIV transmission.

### Pre-exposure prophylaxis with antiretrovirals

Researchers are evaluating the drug tenofovir – a nucleotide reverse transcriptase inhibitor currently approved for the treatment of HIV – in high-risk, HIV-negative individuals to prevent HIV infection. Tenofovir is administered in pill form and is long lasting, slow to generate resistance, and has relatively few side effects. The foundation has provided support for international trials of tenofovir for HIV prevention.

### Vaccines

A preventive HIV vaccine represents the greatest long-term hope for reversing the epidemic, but research progress has been slow. The foundation has contributed $126 million toward research on promising approaches to HIV vaccination. In addition, the foundation has joined with leading research agencies and other funders to form the Global HIV Vaccine Enterprise, a cooperative alliance that aims to accelerate HIV vaccine development through greater collaboration, strategic focus, and resources. Future grantmaking by the foundation in the vaccine field will be guided by the priorities identified in the Vaccine Enterprise's scientific strategic plan [12].

## Conclusion

The Millennium Development Goals (MDGs) recognize the toll that HIV is taking on individuals and societies, as well as the central role that the fight against HIV/AIDS plays in broader efforts to promote human health and international development. History is counting on all of us to realize that the relentless expansion of HIV is not inevitable, and that a focused, concerted response can save millions of lives.
